# Are measurements of patient safety culture and adverse events valid and reliable? Results from a cross sectional study

**DOI:** 10.1186/s12913-015-0852-x

**Published:** 2015-05-02

**Authors:** Per G Farup

**Affiliations:** Department of Research, Innlandet Hospital Trust, N-2819 Gjøvik, Norway

## Abstract

**Background:**

The association between measurements of the patient safety culture and the “true” patient safety has been insufficiently documented, and the validity of the tools used for the measurements has been questioned. This study explored associations between the patient safety culture and adverse events, and evaluated the validity of the tools.

**Methods:**

In 2008/2009, a survey on patient safety culture was performed with Hospital Survey on Patient Safety Culture (HSOPSC) in two medical departments in two geographically separated hospitals of Innlandet Hospital Trust. Later, a retrospective analysis of adverse events during the same period was performed with the Global Trigger Tool (GTT). The safety culture and adverse events were compared between the departments.

**Results:**

185 employees participated in the study, and 272 patient records were analysed. The HSOPSC scores were lower and adverse events less prevalent in department 1 than in department 2. In departments 1 and 2 the mean HSOPSC scores (SD) were at the unit level 3.62 (0.42) and 3.90 (0.37) (p < 0.001), and at the hospital level 3.35 (1.53) and 3.67 (0.53) (ns, p = 0.19) respectively. The proportion of records with adverse events were 10/135 (7%) and 28/137 (20%) (p = 0.003) respectively.

**Conclusions:**

There was an inverse association between the patient safety culture and adverse events. Until the criterion validity of the tools for measuring patient safety culture and tracking of adverse events have been further evaluated, measurement of patient safety culture could not be used as a proxy for the “true” safety.

## Background

Adverse events are common in the health services due to system weaknesses and individual errors [1-3]. It has been estimated that 0.9 – 5.2% of deaths in hospitals are potentially preventable, which corresponds to 1.735, 11.859 and 210.000 - 400.000 deaths per year in Dutch, English and US hospitals respectively [3-5]. Since individual errors are in part inevitable, focus has changed from blaming individuals to system improvements [6]. Various initiatives have been taken to ameliorate the system, such as improving the safety culture, but evidence of the benefit is insufficient [7].

Underreporting and lack of suitable measuring tools make estimates of the true prevalence of preventable adverse events unreliable [5]. The Global Trigger Tool (GTT) developed by Institute for Healthcare Improvement, is an objective and retrospective method of detecting adverse events [8]. It has been presumed that a good patient safety culture is related to patient safety [9,10].

The validity and reliability of the tools used for measurements of patient safety culture and adverse events have been questioned [11-15] and the relation between the safety culture and adverse events needs clarification [14,16-19].

The aim of this cross sectional study was to explore associations between the safety culture and adverse events, and evaluate the validity of the tools used for the measurements.

## Methods

### Hospital structure

Innlandet Hospital Trust has a catchment population of nearly 400,000 inhabitants and consists of six somatic hospitals dispersed over a large area.

### Participants

In 2008/2009, a survey on patient safety culture was performed in the hospital, and one paper based on some of the results from the survey has been published [20]. In this study, two medical departments at two geographical units with significant differences in patient safety culture were selected for a retrospective review of adverse events from the same period. The departments were chosen because of similar functions (except for more patients with malignancies in one of them), representative response rates from all personnel groups, but unequal size and little communication between them. In all, they were judged as best fitted for the comparisons.

### Safety culture

A validated Norwegian version of Hospital Survey on Patient Safety Culture (HSOPSC) designed by Agency for Healthcare Research and Quality (AHRQ) was used for the survey on patient safety culture. The questionnaire has been used worldwide including in the Nordic countries (Norway and Sweden) [21-25]. The US version of the questionnaire with user’s guide is published on the web [26]. HSOPSC consists of 44 questions which are combined to seven dimensions of the safety culture at the unit level, three dimensions at hospital level and four outcome measures. In this study, the overall safety culture at the unit levels and hospital levels were used. Each question is scored from 1 to 5 (5 is best), and a mean score was calculated for each participant. Scores 4 and 5 have been classified as a positive response, and the proportion of positive response was calculated for each participant in addition to the mean score. A proportion of positive response > 75% has been judged as desirable. In addition to the overall safety culture measurements, the participants’ perception of patient safety grade (score: 1 = Failing, 2 = Poor, 3 = Acceptable, 4 = Very Good, 5 = Excellent) and number of events reported per year (score: 1 = No reports, 2 = 1-2 Reports, 3 = 3-5 Reports, 4 = 6-10 Reports, 5 = 11-20 Reports, 6 ≥ 20 Reports) were recorded.

### Adverse events

Institute of Healthcare Improvement has developed the GTT for identifying adverse events in health care institutions. Trained teams perform retrospective reviews of inpatient hospital records for identification of “triggers” which might indicate adverse events, and adverse events are searched for in records with “triggers”. The method determines harm rates, observes changes over time and classifies the harms according to the National Coordinating Council for Medication Error Reporting and Prevention (NCC MERP Index) into the categories E –I; E: Temporary harm to the patient and required intervention; F: Temporary harm to the patient and required initial or prolonged hospitalization; G: Permanent patient harm; H: Intervention required to sustain life: I: Patient death [8,27]. One trained GTT team screened all patient records to avoid differences between teams [12,13]. The GTT and not the self-reported safety outcome dimensions of HSOPSC was used to achieve an independent evaluation of adverse events.

### Variables

In addition to the variables in the HSOPSC questionnaire, the participants’ age (≤30; 31-40; 41-50; 51-60; >60 years of age), gender, profession, and length of service in the department (≤1; 1-5; 6-10; 11-15; 16-20; > 20 years) were noted. The following variables were recorded in the patient records: Gender, age (years), stay in hospital (days), emergency admittance (yes/no), malignancy (yes/no) and Diagnosis-Related Groups (DRG) points. DRG is an official measurement of the service (complexity) to each patient and a tool for reimbursement of the hospital. The patients’ official DRG points were retrieved from the hospital’s administrative system.

### Statistics

In addition to descriptive statistics, comparisons have been performed with exact chi-square tests, chi-square tests for trends (linear-by-linear) when appropriate, and student *t*-test and Mann-Whitney *U*-test for continuous variables with and without normal distribution respectively. Multivariable statistics was performed with linear and logistic regression analyses for continuous and binary outcomes respectively. SPSS version 18 was used for the analyses and p-values below 0.05 were considered as statistically significant.

Based on measurements in this hospital and reviews, the proportion of patients with adverse events in the hospital was assumed to be 9-15% [1,2]. In order to detect a difference in the prevalence rates of adverse events of 10% (prevalence rates 10% and 20% respectively) between the two departments (α = 0.05; 1-β = 0.8) a GTT examination of 200 patient records from each department was planned.

### Ethics

Participation was voluntary, and the survey was performed anonymously. The head of the departments initiated the screening of patient records with the GTT, and the results were anonymized before they were made available for research. The Norwegian Data Inspectorate represented by the Privacy Ombudsman for Research at Oslo University Hospital approved the study.

## Results

### Participants

**Figure 1:**
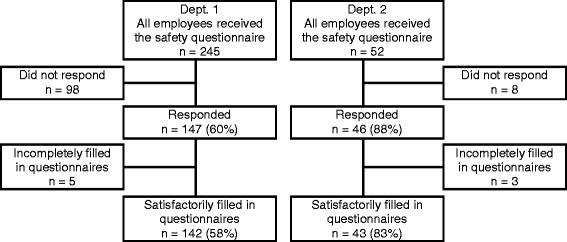
Participants in the study.

In all 245 and 52 employees in departments 1 and 2 respectively were invited to participate in the patient safety survey and 142 (58%) and 43 (83%) had satisfactorily filled in questionnaires and were included in this study. Figure 1 shows the details. Table 1 gives the characteristics of the participants in the two departments. Except for a significantly higher proportion of participants with the profession “other” (mostly administrative personnel) in department 2, there were no significant differences between the participants in the two departments.

**Table 1:** The participants’ characteristics and HSOPSC* scores

**Variables (no of participants)**	**Department 1**	**Department 2**	**Statistics**
Participants’ characteristics			
Male / female (167)	105 (83%)/21 (17%)	33 (80%)/8 (20%)	ns (p = 0.64)
Age (years) (169)			ns (p = 0.09)
≤30	26 (20%)	4 (10%)	
31 – 40	31 (24%)	6 (15%)	
41 – 50	33 (25%)	14 (36%)	
51 – 60	33 (25%)	12 (31%)	
>60	7 (5%)	3 (7%)	
Profession (170)			p < 0.001
physician	21 (16%)	5 (13%)	
registered nurse	92 (71%)	22 (55%)	
auxiliary nurse	16 (12%)	2 (5%)	
other	1 (1%)	11 (28%)	
Length of service (years) (174)			ns (p = 0.86)
≤1	13 (10%	3 (7%)	
1 – 5	35 (26%	15 (37%)	
6 – 10	40 (30%)	8 (20%)	
11 – 15	21 (16%)	6 (15%)	
16 – 20	7 (5%)	2 (5%)	
>20	17 (13%)	7 (17%)	
HSOPSC*			
Unit level – mean scores (SD)	3.62 (0.42)	3.90 (0.37)	p < 0.001
Unit level – mean proportion (%) of positive response (SD)	59.8 (20.7)	71.5 (18.4)	p = 0.001
Hospital level – mean (SD)	3.35 (1.53)	3.67 (0.53)	ns (p = 0.19)
Hospital level – mean proportion (%) of positive response (SD)	37.1 (26.4)	62.6 (27.1)	p < 0.001
Patient safety grade – mean (SD) (154)	3.40 (0.59)	3.79 (0.50)	p = 0.002
No of events reported -mean (SD) (155)	2.2 (1.2)	1.6 (0.9)	p = 0.006

*HSOPSC = Hospital Survey on Patient Safety Culture.

### Safety culture

The safety culture scores were higher in department 2 than in department 1, the differences were statistically significant for all comparisons except for the mean culture score at the hospital level (Table 1). After correction for differences between the participants in the two departments (multivariable linear regression), all differences in safety culture between the departments were statistically significant (all p-values < 0.002; data not shown). The participants in department 2 judged the patient safety in their department as significantly better and reported significantly fewer adverse events than the participants in department 1 (Table 1).

### Adverse events

In all, 272 consecutive patient records were analysed retrospectively with the GTT. Table 2 gives the characteristics of the patients in the two departments and the number of patients with adverse events. The prevalence of adverse events in departments 1 and 2 were 7% and 20% respectively (p = 0.003). Table 3 gives the severity of adverse events in the two departments. Only adverse events that caused temporary harm and required intervention (category E - the least serious ones) were more prevalent in department 2. There were no statistically significant differences between the patients with and without adverse events (Table 4). Table 5 gives the independent predictors of adverse events (logistic regression analyses). Department was the only variable that was significantly associated with adverse events.

**Table 2:** Characteristics of the patients analysed with the GTT* and the number of detected adverse events

**Patients’ characteristics**	**Department 1**	**Department 2**	**Statistics**
No of patients	135	137	
Male (no)	71 (53%)	67 (49%)	ns (p = 0.55)
Age (years)	66 (15)	71 (17)	p = 0.01
Stay in hospital (days)	6.8 (6.6)	4.7 (3.2)	p < 0.001
Emergency admittance (no)	121 (90%)	128 (94%)	ns (p = 0.14)
Malignancy (no)	98 (73%)	18 (13%)	p < 0.001
Diagnosis-Related Groups (points)	0.76 (0.27)	0.52 (0.22)	p < 0.001
Adverse events (no)	10 (7%)	28 (20%)	p = 0.003

The results are given as number (%) and mean (SD).

*Global Trigger Tool.

**Table 3:** The severity of adverse events in the two departments classified according to NCC MERP*

	No AE**	AE** category E	AE** category F	AE** category G	AE** category H	AE** category I	**Sum**
Dept 1	125 (93%)	5 (3.7%)	2 (1.5%)	0 (0%)	0 (0%)	3 (2.2%)	135 (100%)
Dept 2	109 (80%)	21 (15%)	3 (2.2%)	1 (0.7%)	0 (0%)	3 (2.2%)	137 (100%)

The results are given as number of patients with percentage in brackets.

*National Coordinating Council for Medication Error Reporting and Prevention Classification of adverse events: E: Temporary harm to the patient and required intervention; F: Temporary harm to the patient and required initial or prolonged hospitalization; G: Permanent patient harm; H: Intervention required to sustain life; I: Patient death.

**AE = Adverse event.

**Table 4:** **The characteristics of patients with and without adverse events**

	**Patients without adverse events**	**Patients with adverse events**	**Statistics**
No. of patients	234	38	
Male	114 (49%)	24 (63%)	ns (p = 0.12)
Age (years)	68 (17)	70 (14)	ns (p = 0.50)
Stay in hospital (days)	5.7 (5.5)	6.0 (3.8)	ns (p = 0.77)
Emergency admittance	213 (91%)	36 (94%)	ns (p = 0.78)
Malignancy	105 (45%)	11 (29%)	ns (p = 0.08)
Diagnosis-Related Groups (points)	0.64 (0.28)	0.61 (0.26)	ns (p = 0.48)

The results are given as number (%) and mean (SD).

**Table 5:** **Independent predictors of adverse events (logistic regression analyses)**

**Predictor**	**OR**	**95% CI of OR**	**Statistics**
Department 2	3.64	1.30 – 10.24	p = 0.014
Sex (female)	0.53	0.25 – 1.09	ns (p = 0.09)
Age	1.00	0.98 – 1.03	ns (p = 0.84)
Stay in hospital (days)	1.04	0.96 – 1.11	ns (p = 0.34)
Emergency admittance	0.77	0.16 – 3.60	ns (p = 0.74)
Malignancy	1.15	0.42 – 3.15	ns (p = 0.79)
Diagnosis-Related Groups (points)	1.43	0.31 – 6.70	ns (p = 0.65)

The results are given as Odds Ratio (OR) with 95% confidence intervals (CI) and the statistics as p-values and significance (ns = not statistically significant).

## Discussion

The finding of more adverse events in the department with the best safety culture was unexpected, and questions the reliability and validity of the tools used for measuring the patient safety culture and the adverse events.

Except for the culture at the hospital level in department 1, the overall safety culture in the departments was satisfactory. In a database with results from 1128 hospitals and 567,703 hospital staff respondents reported by Agency for Healthcare Research and Quality, the mean positive response rates at the unit and hospital level were 64.7% and 58.3% respectively, and values outside ±5% were judged as statistically significant [28]. Compared with this database, the positive response rate at the hospital level in department 1 was unsatisfactory (37.1%) and the positive response rate at the unit level in department 2 was very good (71.5%). The low response rate in department 1 might have, for unknown reasons, selected participants who were critical to the culture.

The high response rates in department 2 and the participants’ favourable responses to the HSOPSC questionnaire might reflect the participants’ motivation for high-quality work and adherence to procedures and requests. They might unconsciously have given the “correct” answers. They also judged the patient safety as better and reported fewer adverse events than participants in department 1 despite the finding of more adverse events in department 2. The Kruger-Dunning effect described as “difficulties in recognizing one’s own incompetence lead to inflated self-assessments” could explain the inverse association between the culture and adverse events [29].

The prevalence of adverse events differed significantly between the departments. The GTT focuses mainly on adverse events in surgical departments and emergency settings. The evaluation for use in medical departments has not been equally good. Compared to the prevalence of adverse events published from other hospitals, which has been in the order of 4-17%, the prevalence in department 2 was unexpectedly high [1-3,30]. The estimates of adverse events with the GTT vary between analysing teams and depend probably on the patient record system [12,13]. In this study, one team analysed all patient records and the departments used the same electronic patient record system. The GTT retrieves only recorded adverse events. A higher awareness of adverse events in department 2 might have resulted in a better recording of minor events, which could explain the differences between the departments.

The safety culture is only one out of 20 factors mentioned as influencing clinical practice, and the association between the patient safety culture and adverse events seems to be marginal [10,16-18]. Studies and reviews conclude that research problems are related to definition and observation of adverse events, question the implication and generalizability of the results, and doubt the causal relationship between the culture and adverse events [16-18].

The psychometric properties of the tools for measuring patient safety culture and adverse events are of vital importance for the interpretation of the results, but not all psychometric properties of these questionnaires have been satisfactorily documented [14,31]. In addition, to extend their use outside the context (geographical region and healthcare system) in which they were developed demands new validations [15]. Criterion validity (the relation between the measurement and some other variable) and responsiveness (the ability to detect changes within groups) are important properties that have not been satisfactorily studied [14,31-33].

Patient safety (harm) and not “culture” is the most important criterion to be predicted by the patient safety culture surveys. Studies often report self-reported patient safety outcomes such as procedures and behaviour, and not independent measurements of adverse events [17,32,33]. This study demonstrated that the self-reported evaluation of patient safety differed from independently measured adverse events. The department with highest self-appraised patient safety had the highest prevalence of adverse events. The results indicate poor criterion validity of the measurement of patient safety culture. A review of psychometric properties of health-related questionnaires concluded that criterion validity was rarely reported [31]. Reviews of the psychometric properties of patient safety culture have reported no or only a moderate association between the culture and patient outcomes, and are uncertain about the causal relations and the responsiveness [14,17,18,33]. Studies claiming satisfactory criterion validity have used inappropriate criteria closely associated with measurement of the culture such as data collected by a questionnaire to the same personnel about working behaviour, involvement in safety activities, micro accidents, minor injuries, near-misses, compliance with safety rules and procedures, safety initiatives, safety compliance, safety participation, risk taking, rule breaking etc. [17,32]. In this study, the recording of the patient safety culture and the adverse events were completely independent of each other. The study indicates that comparisons of the patient safety culture across departments do not allow conclusions about differences in the “true” safety in the departments. This study and critical reading of the literature show that the criterion validity of surveys on patient safety culture is insufficiently documented for patient harm [34]. Therefore, surveys on the patient safety culture should not be used as proxies of the “true” patient safety until the criterion validity is better documented.

The GTT aims at measuring the prevalence of harm and changes over time [8]. The method has been judged as both appropriate and inappropriate for the purpose [11-13,30,35]. Most triggers are related to surgical procedures, and most evaluations have been performed in surgical and emergency units. The triggers in the Norwegian version of the GTT have never been evaluated for medical departments. The results will probably depend on the medical record system and the way events are recorded. Since the GTT never detects all adverse events and the proportion detected is unknown, the results do not indicate the true prevalence of adverse events. An important weakness is the large inter-rater variability. Studies have shown a variance in Cohen Kappa coefficients from 0.26 to 0.77 and in the prevalence of adverse events between the teams from 27.2. to 99.7 per 1000 hospital days, and that only 31% of adverse events were identified by two different teams [11-13,35]. The random error in these studies was large, and the sensitivity for detection of adverse events for a local team was 49% of the prevalence of an expert team [11,13]. Conclusions about the usability of the GTT vary enormously from recommendations to avoidance [11-13]. The results unveil major problems related to registration of adverse events, and demonstrate that the GTT probably is inappropriate for comparisons between units, departments, and hospitals and as an indicator of the true prevalence of adverse events. The GTT might be suitable for tracking changes in adverse events over time given that the measurements are performed in one single unit, by the same experienced team, with the same patient record system and a stable staff recording the events in the same way. This use of the GTT needs evaluation in studies with a focus on intra-rater reliability and responsiveness. The GTT is, nevertheless, better than self-reported measurements of adverse events [33].

### Strengths and limitations

The rather small size of this study and the low response rate in one department reduce the reliability and render new and larger studies necessary. Valid information about associations between patient safety culture and adverse events requires studies with more participants in more than two departments, and the registration of adverse events over longer periods. Nevertheless, the unexpected result in this study calls attention to the lack of knowledge related to the measuring tools. It strengthens the study that the measurement of the culture and registration of adverse events were performed independently of each other, that one trained team performed all the GTT measurements, and that the departments were parts of the same hospital trust with the same patient record system and many common routines.

The number of patient records screened with the GTT was lower than planned in the protocol. Since the difference in adverse events between the departments was larger than presumed, this has probably not influenced significantly on the results.

## Conclusions

The inverse relationship between patient safety culture (measured with HSOPSC) and adverse events (measured with the GTT) seen in this study indicates that the validity (particularly the criterion validity) and reliability of tools for measuring patient safety culture and tracking of adverse events need further evaluation and that results from such measurement should be interpreted with caution. To use the patient safety culture as a proxy for the “true” patient safety must be avoided until more information about the criterion validity is available.
